# Immunotherapy in gastrointestinal cancers: current strategies and future directions – a literature review

**DOI:** 10.1097/MS9.0000000000002757

**Published:** 2025-01-09

**Authors:** Serene El Fil, Olivier Uwishema, Aisha Rizwan Ahmed, Tanya Ratnani, Ameen Rupani, Sarah Mshaymesh

**Affiliations:** a Department of Research and Education, Oli Health Magazine Organization, Research and Education, Kigali, Rwanda; b Department of Natural Sciences, School of Arts and Sciences, Lebanese American University, Beirut, Lebanon; c Jinnah Medical and Dental College, Karachi, Pakistan; d Chhattisgarh Institute of Medical Sciences, Bilaspur, India; e International Higher School of Medicine, Bishkek, Kyrgyzstan; f Division of Natural Sciences, Faculty of Sciences, Haigazian University, Beirut, Lebanon

**Keywords:** gastrointestinal (GI) cancer, immune checkpoint inhibitors (ICIs), immunotherapy, personalized treatments, predictive biomarkers

## Abstract

**Introduction:**

The National Cancer Institute defines the disease of “cancer” as a group of disorders in which aberrant cells proliferate uncontrollably and have the potential to infiltrate neighboring tissues. It is well established that cancer remains a significant etiology contributing to worldwide mortality. Gastrointestinal (GI) neoplasms are a type of cancer that affects the digestive system and adds to the total cancer burden. Conventionally, several therapies have been employed, such as radiation and chemotherapy; nevertheless, their adverse effects have prompted the need for an improved therapeutic alternative. Immunotherapy thus became a notable medium of treatment for several malignancies, including tumors of the GI tract.

**Aim:**

This comprehensive review seeks to provide insight on future directions and prospective therapies under development, as well as information regarding the present strategies utilized to mitigate one of the primary forms of cancer, GI cancer.

**Methods:**

A detailed analysis of the existing literature on GI cancers has been conducted. Several databases were employed to gather this information, mainly PubMed/MEDLINE. Different aspects of the disease were considered when searching the databases to provide a comprehensive review of the current and future strategies being incorporated to mitigate the negative consequences of this disease.

**Results:**

Many strategies are being used currently, and some are still under development. These comprise the usage of immune checkpoint inhibitors (ICIs), cytokine therapy, cancer vaccines, oncolytic viruses, and adoptive cell therapy. For instance, various monoclonal antibodies have been developed to inhibit the immunomodulatory effects of programmed death-1 and programmed death-1 ligand. There are also results of several clinical trials showing significant benefits and many changes are introduced to make the best of these strategies and minimize the challenges to group sizes. These challenges include overcoming the tumor’s immunosuppressive environment, finding suitable predictive biomarkers, and reducing the adverse effects. Additionally, several novel immunotherapeutic approaches, such as chimeric antigen receptor T-cell (CAR-T) therapy, are being studied. In 2017, the US FDA approved the use of two CAR-T therapies, which marks a major milestone following extensive research and clinical trials. New approaches such as toll-like receptor-directed and helminth-based immunotherapies are being developed for the treatment of GI cancers as well. These therapies, along with targeted treatments, represent the future of immunotherapy in GI cancers.

**Conclusion:**

Immunotherapy plays a significant role in the different types of GI cancers. However, optimizing these treatments will require overcoming barriers such as immune resistance, minimizing side effects, and improving the selection of patients through biomarkers. Continued research into these novel therapies and the mechanisms of immune modulation will be key to maximizing the therapeutic benefits of immunotherapy in the future.

## Introduction

Cancer is a generic word for a broader group of diseases that can alter any part of the human body, associated terminology with it are malignant tumors and neoplasms[1]. One key feature of cancer is the accelerated production of abnormal cells that originate beyond their normal ability, which can occupy neighboring parts of the body and spread to other organs, the former process known as metastasis, the primary cause of death by cancer[1].

Cancer is the foremost cause of mortality around the world, causing close to 10 million mortalities or nearly one in six deaths[1]. The most common are that of breast, lung, colon, rectum, and prostate cancers. One-third of cancer mortalities are accountable to the usage of tobacco, high body mass index, consumption of alcohol, low intake of fruits and vegetables indicating reduced dietary fiber, and physical inactivity[1]. Early detection of many cancers may be managed early with cost-effectiveness[1].

The gastrointestinal (GI) tract is a 25-foot-long pathway that extends from the mouth to the anus[2]. Food eaten passes as a bolus through the esophagus and is processed in the stomach and small intestines to extract nutrients[2]. Ultimately, the waste is removed from the body through the colon and rectum[2]. Sometimes, a tumor may form in one of these organs post-change in DNA causing abnormal cells to undergo mitotic division[2]. This multifaceted in nature.

The most common types of GI cancers are esophageal, gastric, colorectal, pancreatic, and liver[2]. Other types are much less common, including those belonging to neuroendocrine and gastrointestinal stromal tumors alongside anal cancer[3].

GI malignancies contribute to one in four cancer cases and one in three cancer mortalities worldwide[4]. There is a significant change in incidence and mortality rates globally[4]. For example, the incidence of gastric cancer (GC) has decreased but colorectal cancer (CRC) incidence has increased in formerly low incidence rate regions[4]. Additionally, the incidence of liver and pancreatic cancer has risen in certain high-income regions[4]. Prevention strategies include control of tobacco, alcohol, and obesity, immunizing the population against hepatitis-B virus infection, and screening for CRCs[4].

Data from three databases, GLOBOCAN, Cancer Incidence in Five Continents, and the World Health Organization mortality database, demonstrated that the incidence and mortality trends for GI malignancies differed by geography[5]. In 2018, Asia accounted for the bulk of new cases (63%) and deaths (65%), with Europe and North America accounting for 26% and 23%, respectively[5]. Asia had higher rates of esophageal, gastric, and liver cancers, while Europe and North America had higher rates of colorectal and pancreatic cancers[5].

In the realm of oncological intervention, various methods such as conventional radiotherapy and chemotherapy have been employed. However, these come with their own set of limitations. The limitations of conventional radiotherapy, chemotherapy, or both include the following: inaccurate and unsafe radiation delivery, lack of sensitivity, and the development of drug resistance during treatment^[6,7]^. Other limitations involve reduced drug uptake, increased drug export, inactivation of the effective agent within cells, changes to the molecular target, increased activity of the target pathway, and appearance or stimulation of alternative pathways^[6,7]^. Additionally, enhanced repair of drug-induced modifications in the target molecules and activation or inhibition of intracellular signaling pathways can result in an imbalance between apoptosis and survival of tumor cells^[6,7]^. Furthermore, differences among primary malignant gastrointestinal tumors, the diversity of tissues from which metastatic cells may access the gut, inter-individual variability, and the potential for sensitivity or resistance to change during tumor evolution further complicate treatment^[6,7]^.

Immunotherapy is another type of cancer treatment. It uses substances made by the body or synthetically to boost the immune system and assist the body in locating and destroying cancer cells[8]. Immunotherapy may treat many different types of cancer, alone or in combination with chemotherapy and/or other cancer therapeutic modalities[8]. In contrast to traditional therapies, immunotherapy is considered a targeted therapy, employing medication that targets cancer-specific genes, proteins, or the tissue environment that aids tumor growth and survival[8].

Cancer immunotherapy emphasizes the importance of understanding tumor immunology – particularly the roles of tumor antigens and the immunosuppressive tumor microenvironment[9]. Many novel immunotherapy agents have been developed that effectively combat cancer. While many cytokine-based approaches and their derivatives have become treatments deemed the standard-of-care for a variety of malignancies, other immunotherapy approaches, such as most cancer vaccines and cell-based approaches, remain experimental[9]. Fortunately, many novel immunotherapeutic agents are being researched and tested in clinical trials, which will hopefully provide new effective treatments for patients living with relapsed or refractory malignancies.

This review aims to comprehensively analyze the current landscape of immunotherapy strategies for GI cancers. Furthermore, we will look toward the future, examining the potential advancements for immunotherapy in GI cancers. This review seeks to provide health care professionals and researchers with a current and insightful analysis of this rapidly evolving field.

## Current strategies of immunotherapy for GI cancers

Immunotherapy-based regimens have revolutionized the management of recurrent or metastatic GI cancers. Recent evidence has identified many tumor cells escape cellular immunoediting using various mechanisms involving immune checkpoint proteins, cytokines, and antigens. Multiple novel approaches are utilized to inhibit immunomodulation in GI cancers^[10-12]^.

### Immune checkpoint inhibitors

The stimulatory or inhibitory signals generated by the interaction of programmed death-1 (PD-1) and cytotoxic T-lymphocyte antigen-4 (CTLA-4) with their ligands found on the surface of antigen-presenting cells (APC) or tumor cells are blocked by immune checkpoint inhibitors (ICIs)[10].

**Figure 1.**
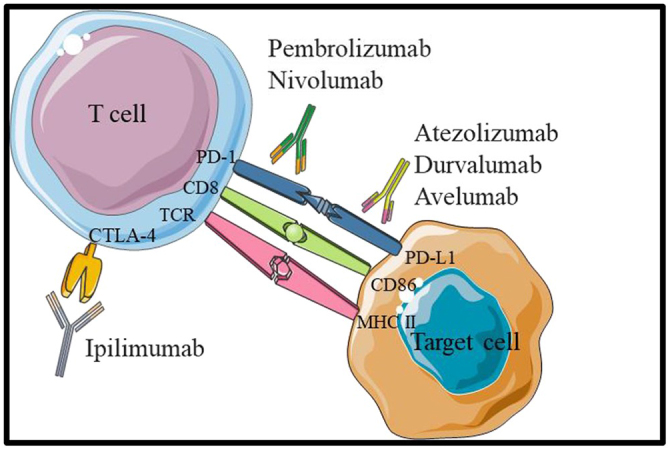
Binding between T-cells and cancer cell.

PD-1 is present on the surface of lymphoid cells. PD-1 attachment with its ligand (programmed death-1 ligand, PD-L1) on tumor cells results in the deactivation of previously activated lymphoid cells (Fig. 1) (further discussed in future directions)[10]. Various monoclonal antibodies have been developed to inhibit the immunomodulatory effects of PD-1 and PD-L1 (Table 1; Supplementary Table 1, http://links.lww.com/MS9/A667). Similarly, CTLA-4 is found on the cell surface of both CD4-positive and CD8-positive lymphocytes and binds to co-stimulatory factors on APCs[10]. This interaction decreases interleukin-2 (IL-2) production and T-cell proliferation[10] (Table 5).

**Table 1:** Approved PD-1 in gastric cancer as per National Comprehensive Cancer Network guidelines[16]

Agents	Indication
Pembrolizumab	Unresectable or metastatic MSI-H or dMMR solid, TMB-H solid tumors, MSI-H/dMMR or TMB-H gastroesophageal tumors
Nivolumab	Advanced or metastatic gastric cancer
Dostarlimab-gxly	MSI-H/dMMR gastric tumors

dMMR, mismatch repair deficient; MSI-H, microsatellite instability-high; PD-1, programmed death-1; TMB-H, tumor mutational burden-high.

A meta-analysis of randomized controlled trials of patients diagnosed with metastatic gastric/gastroesophagojejunal adenocarcinoma revealed that the addition of PD-1 inhibitors and not PD-L1 inhibitors to standard treatment has superiority in terms of overall survival (OS) and progression-free survival (PFS)[13]. Furthermore, in patients with microsatellite instability-high (MSI‐H) disease, adjuvant ICIs have benefits (further discussed in future directions)[13].

Various meta-analyses have demonstrated the superiority of adjuvant ICIs over multiple combined modalities in terms of OS and PFS^[13,14]^. However, treatment-related adverse events were more prevalent in ICI groups[14]. Another meta-analysis revealed that ICIs alone or in combination with chemotherapy have benefits in OS in Asians, patients with microsatellite instability-high (MSI-H), PD-L1, and tumor mutational burden (TMB-positive) advanced GC[15].

### Cytokine therapy

Cytokines are molecular messengers of the immune system enabling immune cells to communicate over short distances. Cytokine therapy aids the host’s immune cells to target and destroy cancer cells. Clinical research has focused on utilization of several cytokines for the treatment of GCs[17] (Table 2).

**Table 2:** Roles of cytokines in immunity^[17,18]^

Roles of cytokines in immunity
Induction of immunological checkpoints Induces the secretion of inhibitory factors such as IL-10 and TGF-β Induces the expression of inhibitors such as TIM Induces activation of immune-dampening cells, including Tregs and MDSC Induces activation of intracellular suppressors of cytokine signaling (CIS and SOCS) proteins that terminate the CD4 T-cell immune response Induces the proliferation of activated NK cells

CD, cluster of differentiation; CIS, cytokine-inducible SH2-containing protein; IL-10, interleukin-10; MDSC, myeloid-derived suppressor cells; NK, natural killer cell; SOCS, suppressors of cytokine signaling; TGF-β, transforming growth factor beta; TIM, triosephosphate isomerase; Tregs, regulatory T-cells.

Clinical research has pinpointed several cytokines, including interferon-alpha (IFN-α), IL-2, IL-12, IL-15, and IL-21, for potential use in cancer management. Nevertheless, none of these cytokines have gained approval as a primary therapy for managing GCs[19]. The combination of cytokine therapy and chemotherapy enhances immune cell functionality and quality of life, and extends survival period with fewer adverse events^[20,21]^. Similarly, a clinical trial has demonstrated enhanced tumor-infiltrating T-cell activity in GCs, preventing surgery-induced lymphopenia, and improving survival outcomes through subcutaneous IL-2 administration preoperatively^[22,23]^.

### Cancer vaccines

Cancer vaccine is another novel immunotherapy in GI cancers, activating the immune response to tumor cells[24]. To date, numerous cancer vaccines have been developed (Table 3). The clinical trials have demonstrated that peptide vaccines are well-tolerated, showing delayed hypersensitivity and eliciting an approximately 75% positive response from cytotoxic T-lymphocytes (CTLs)^[25,26]^. Similarly, gastrin-17 diphtheria toxoid, an immune-conjugated vaccine, combined with chemotherapy has improved survival outcomes in advanced GC and gastroesophageal cancer patients[27]. A recent study has introduced a vaccine design approach aimed at creating an *in silico* vaccine against CRC[28]. The strategy involves targeting *Fusobacterium nucleatum*, a significant bacterial pathogen linked to the initiation and advancement of tumors. The vaccine specifically targets the *F. nucleatum* membrane protein known as fibroblast activation protein-2 (Fap2), which facilitates bacterial attachment to colon cells and initiates the recruitment of immune cells and tumorigenesis. By incorporating B-cell and T-cell epitopes of Fap2, the vaccine seeks to bolster both cell-mediated and humoral immune responses. This proposed vaccine shows promise as a therapeutic intervention for *F. nucleatum*-induced human CRC[28].

**Table 3:** Types of cancer vaccines and stimulus

Vaccine type	Stimulus
Cell-based vaccines	Activation of innate immune cells
Dendritic cell vaccines	
Whole cell vaccines	
Nucleic acid-based vaccine	
RNA vaccines	
DNA vaccines	
Induced pluripotent stem cell-based vaccine	Recognition of vaccine components by immune cells
*In situ* cancer vaccine	Activation of immune cells in tumor microenvironment
Microbial vector vaccines:	Direct activation of immune response
Viral-based vaccines	
Bacteria-based vaccines	
Peptide vaccine	Recognition of peptide antigens
Exosome-based vaccine	Activation of dendritic cells

### Oncolytic viruses

Oncolytic virotherapies (OVTs) selectively target and destroy cancer cells while inducing a systemic antitumor immune response, transforming unresponsive “cold” tumors into responsive “hot” tumors^[29,30]^. OVTs comprise a broad diversity of viruses both naturally cancer selective or can be genetically engineered^[31,32]^ (Table 4). The effectiveness of OVTs depends on several factors, such as virus/host interaction, degree of innate immunity and inflammation induced, types of virus-induced cell death, tumor physiology and phenotype, cell surface immune markers, and expression of immunosuppressive factors[30]. Genetic engineering has enabled the manipulation of pathogenic viruses for safety purposes and the development of more personalized and targeted therapies[32].

**Table 4:** Types of oncolytic viruses^[31,32]^

Natural cancer selective	Genetically engineered
Parvoviruses, myxoma virus, Newcastle disease virus, reovirus, and Seneca valley virus	Measles virus, poliovirus, vaccinia virus, adenovirus, herpes simplex virus, and vesicular stomatitis virus

**Table 5:** Ongoing clinical trials on immunotherapy in gastrointestinal cancer

Agents	NCT number
PD-1 inhibitors	
Nivolumab	NCT02872116, NCT03647969, NCT02743494, NCT03662659
Pembrolizumab	NCT03221426
CTLA-4 inhibitors	
Ipilimumab	NCT02872116, NCT03647969
PDL-1 inhibitors	
Atezolizumab	NCT03421288
Durvalumab	NCT04157985
Avelumab	NCT04157985
Oncolytic viral therapy	
Adenovirus therapy	NCT04111172, NCT03740256, NCT03544723
Herpes simplex virus type 2 strain HG52	NCT03866525
Adoptive cell therapy	
Epithelial cell adhesion molecule	NCT03563326, NCT02725125
Human epidermal growth factor receptor-2	NCT04650451, NCT03740256
Carcinoembryonic antigen (CEA)	NCT04348643
Claudin18.2	NCT04581473, NCT04404595
Glypican-3	NCT03198546

CTLA-4, cytotoxic T-lymphocyte antigen-4; PD-1, programmed death-1; PDL-1, programmed death-1 ligand.

Jun *et al*. reveal that a novel genetically engineered vaccinia virus carrying the human sodium iodide symporter (hNIS) gene, GLV-1h153, can efficiently regress gastric tumors and allow deep-tissue imaging[33]. Experimental GI cancer models have shown that OVT administration enhances antitumor immunity and prolongs survival, but its safety and efficacy in humans remain inconclusive^[34-36]^. Ongoing human clinical trials are listed in Table 5.

### Adoptive cell therapy

Immune cells are isolated and collected via leukapheresis, genetically modified, expanded *ex vivo*, and administered back to the patient[37]. The types of adoptive cell therapy (ACT) include tumor-infiltrating lymphocyte therapy, engineered T-cell receptor therapy, chimeric antigen receptor T-cell therapy (CAR-T) (further discussed in future directions), CAR-natural killer (NK) therapy, CAR-NK T therapy, CAR-macrophage (M) therapy, and CAR-γδT therapy[37].

A phase 1 clinical trial revealed carcinoembryonic antigen (CEA) CAR-T cells are well-tolerated in patients with CEA + colorectal liver metastasis[38]. However, first-generation CAR-T is associated with respiratory toxicity[39]. Clinical trials are still ongoing to evaluate the safety and efficacy of ACT (Table 5).

## Challenges in immunotherapy for GI cancers

While immunotherapy offers a promising new avenue for treating GI cancers, several hurdles must be overcome to maximize its effectiveness.

### Overcoming tumor immunosuppression

One major challenge lies in overcoming the tumor’s immunosuppressive microenvironment.

Due to its unique mechanisms of action, it has been found that the mechanisms of immunotherapy resistance are different from those of conventional chemotherapy, and this requires a significant challenge that needs to be addressed[40]. These mechanisms are “complex and heterogeneous” and constitute a combination of many genes and metabolism^[40,41]^ and can be divided into two different types: intrinsic and extrinsic^[40-42]^.

Intrinsic mechanisms are those that include the alterations in antitumor immune response pathways and changes in signaling pathways in tumor cells which in turn lead to the formation of an inhibitory immunosuppressive microenvironment^[40,41]^. On the other hand, extrinsic factors mostly include the ones that are linked to the local tumor microenvironment^[40,41]^.

Disabling these mechanisms is crucial to ensure a successful immunotherapy treatment and future research should focus on the strategies to overcome this resistance. One suggested strategy is developing combination therapies that can work to target these different resistance mechanisms and thus increase the efficiency of the treatment for patients[42].

### Identifying predictive biomarkers

Another challenge is identifying reliable and new biomarkers of GI cancers to predict which patients will benefit from immunotherapy and this allows them to choose the suitable therapeutic option and thus improve the outcomes^[43,44]^.

In order for a biomarker to be valid, it should display several characteristics such as being present in high amounts in patients diagnosed with cancer while being absent or present in low quantities in unaffected patients[43], being easily measured or evaluated^[43,45]^, and being able to provide information about the cancer in the patient^[43,45]^.

A special type of biomarkers is known as “predictive biomarkers,” and these allow the prediction of the response that a patient will give for a certain treatment and thus define the subpopulation that will benefit from that specific therapy[43].

Understanding the importance of these biomarkers is crucial and can change the staging of many GI cancers[45]. Additionally, it is essential that clinicians understand the molecular biology behind GI cancers as they will be requesting biomarker testing as a part of the patient screening[45]. Moreover, it is important to note that strategies for combining more than one biomarker are promising and can help improve the accuracy of the development of the treatment[44].

For instance, new research has pinpointed several crucial genes linked to GI cancers, underscoring their potential as targets for therapy and as biomarkers. In CRC, the COL11A1 gene is excessively expressed and contributes to tumor advancement by upregulating other genes like THBS2, COL10A1, COL5A2, and COL1A2, thereby driving neoplasia[46]. Its overexpression is tied to poor overall and disease-free survival, making it a promising biomarker for assessing CRC prognosis and a target for therapeutic intervention[46]. In GC, Fam198b has been associated with tumor advancement and drug sensitivity, working through the Phosphatidylinositol 3-Kinase/Protein Kinase B (PKB)/B-Cell Lymphoma 2 (PI3K/AKT/BCL-2) signaling pathway[47]. Its suppression significantly hinders tumor cell proliferation and migration, positioning Fam198b as a promising biomarker and a therapeutic target[47]. Furthermore, RNF180 is being investigated as a new tumor suppressor, with potential clinical applications as a biomarker in GC[48]. Together, these genes, among others, present important avenues for advancing GI cancer treatment, particularly in the context of immunotherapy, where targeting specific molecular pathways could improve patient outcomes and customize treatment strategies.

### Adverse effects

Finally, adverse effects associated with immunotherapy are a concern.

Most patients who receive treatment – particularly anti-CTLA-4 therapy – suffer from aphthous ulcers, esophagitis, gastritis, diarrhea, and colitis^[49,50]^.

Thus, it is important that physicians familiarize themselves with these effects and remain in touch with the agent’s providers in order to reduce these unwanted results[49].

## Future directions of immunotherapy for GI cancers

### Novel immunotherapeutic approaches

#### Chimeric antigen receptor T-cell therapy

Chimeric antigen receptor T-cell treatment (CAR-T) involves modifying T-cells in such a way that they eventually can identify particular tumor cell surface antigens without needing the major histocompatibility complex[51].

In the year 2017, the US FDA approved the usage of two CAR-T cell treatments, following years of extensive medical and laboratory research as well as targeted clinical trials^[52,53]^.

Recently, certain laboratories have attempted to utilize EphA2, a protein that is expressed on the surface of many cancer cells. CAR-T cells that recognize EphA2 have shown the ability to recognize and assault esophageal squamous cell carcinoma (ESCC) *in vitro*[54]. These results provide a new direction for ESCC immunotherapy in the future[54]. Coincidentally, liver and other GI malignancies have been successfully treated with CAR-T cell therapy.

Moreover, the human epidermal growth factor receptor-2 (HER2) gene is crucial for function in the incidence and progression of GC, which strongly produces the protein p185[55]. Since p185 is negatively expressed in healthy individuals, it may be a prime candidate for CAR-T cell-based anticancer therapy[55].

Additionally, it has been discovered that CAR-T cell treatment for GI tumors can effectively target CEA, a sensitive tumor biomarker[55]. As a result, many studies have been conducted on the management of various malignancies, including pancreatic cancer, liver cancer, and cholangiocarcinoma. The integration of CAR-T cell therapy with targeted treatments, including those that target CEA, HER2, Glypican-3, Mucin 1 (MUC1), cluster of differentiation (CD133), epithelial cell adhesion molecule, or epidermal growth factor receptor (EGFR) has demonstrated significant promise in combination treatment^[56-59]^.

#### Microsatellite instability-high

Microsatellite instability-high (MSI-H), a predictive predictor of immunotherapy response rates, is frequently brought on by the MMR gene deficiency.

The effectiveness of ICIs in solid tumor patients appears to be associated with MSI, according to mounting data. The phase III KEYNOTE062 trial found that pembrolizumab with chemotherapy did not show a better median OS compared to chemotherapy alone in patients with PD-L1 combined positive score (CPS) of 1 or greater or PD-L1 CPS of 10 or greater[60]. This trial was conducted as the first-line therapy for advanced GC.

A comparison of the median overall survival (mOS) of patients with MSI-H tumors revealed that pembrolizumab with chemotherapy outperformed treatment with chemotherapy alone in terms of survival benefit[60].

It is very critical that MSI-H and PD-L1 are not confused together. While both are important biomarkers for selecting patients, PD-L1 only measures the number of cancer cells that can block the immune response when bound to PD-1. However, MSI-H reveals a high number of mutations due to MMR. In patients with MSI-H tumors, regardless of their PD-L1 level, adding pembrolizumab to chemotherapy significantly improved OS compared to chemotherapy alone. Thus, MSI-H has emerged as a stronger biomarker, especially for patients with GC.

#### Immunotherapy combined with VEGF/VEGFR inhibitors

**Figure 2.**
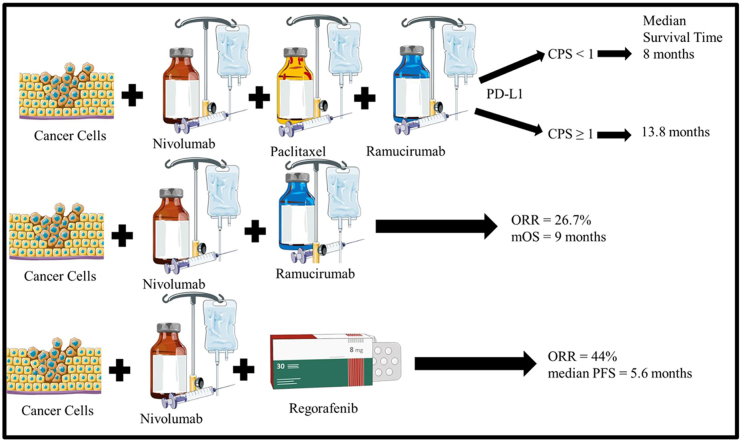
PD-L1 expression and efficacy of Nivolumab-based combination therapies for second-line advanced GC.

Promising clinical activity was observed in a multicenter phase I/II study of nivolumab with paclitaxel plus ramucirumab (Supplementary Table 1, http://links.lww.com/MS9/A667) as a second-line therapy[61]. Compared to patients with lower PD-L1 levels (CPS < 1), those with higher levels (CPS ≥ 1) had a longer median survival time (13.8 vs. 8.0 months) (Fig. 2). In a different phase I/II trial (NIVORam), the objective response rate (ORR) was 26.7% and the mOS was 9.0 months when nivolumab was the only medication used in conjunction with ramucirumab as a second-line treatment for advanced GC[62] (Fig. 2). No direct comparison between the two aforementioned clinical studies can be established because PD-L1 expression in patients in the NIVORam study was not assessed; however, it is plausible that paclitaxel may be omitted as a second-line medication. Nivolumab also demonstrated a strong response rate (ORR: 44%) and median PFS (5.6 months) when paired with regorafenib[61] (Fig. 2/Supplementary Table 1, http://links.lww.com/MS9/A667).

Durvalumab plus ramucirumab also demonstrated safety by the established characteristics of the individual therapies. These findings suggest that vascular endothelial growth factor/vascular endothelial growth factor receptor (VEGF/VEGFR) inhibitor combinations may be useful treatment plans for advanced GC.

**Figure 3.**
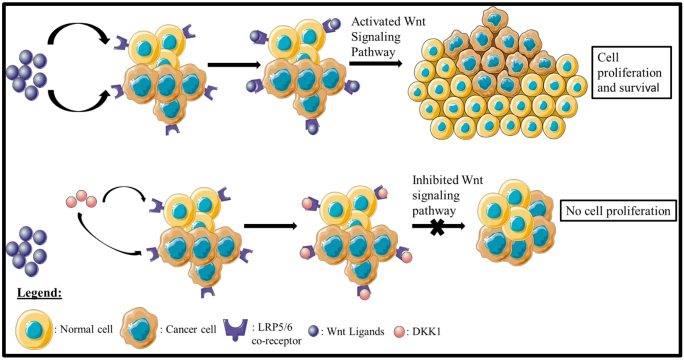
Wnt signaling pathway regulation.

In addition to VEGF/VEGFR, ICIs may be used in conjunction with other targeted treatments. A secreted antagonist, Dickkopf-1 (DKK1), binds to the Wnt co-receptor LRP5/6 and causes cells to become desensitized to common Wnt ligands[63] (Fig. 3). DKK1 thus inhibits the Wnt signaling pathway, which is involved in cell proliferation and survival and is abnormally activated in cancer cells making cells less responsive to these signals.

Patients with advanced GC were treated with pembrolizumab and a neutralizing antibody against DKK1 in an early-stage trial called DKN-01[64]. DKK1 high patients showed an objective reaction of 50%, but DKK1 low patients showed no objective response. Additionally, DKK1 high patients showed prolonged PFS and OS.

#### Dual ICI strategy

For GC, a combination of anti-PD-1 and anti-CTLA-4 has also been investigated. Nivolumab was investigated as monotherapy or in combination with ipilimumab (Supplementary Table 1, http://links.lww.com/MS9/A667) in patients with advanced or metastatic solid tumors in the CheckMate-032 trial[65]. The OS of these two groups was comparable (6.9 vs. 6.2 months) for metastatic and chemotherapy-refractory esophagogastric cancer, despite the combination group (nivolumab 1 mg/kg + ipilimumab 3 mg/kg) having a higher ORR than the nivolumab monotherapy group (24% vs. 12%)[65]. Nonetheless, it appears that combination therapy is more beneficial in the PD-L1-positive and MSI-H categories (18-month survival: 50% for the combination group vs. 13–29% for the nivolumab monotherapy group).

It is anticipated that nivolumab in conjunction with ipilimumab will take center stage as a third-line treatment for GC.

For MSI-H advanced GC or esophagogastric junction cancer, a phase II study (NO LIMIT) combining nivolumab and low-dose ipilimumab is now being conducted as the first-line therapy[65].

#### TLR-directed immunotherapy

Toll-like receptors (TLRs) are crucial components of the innate immune system. They function as pattern recognition receptors that detect pathogen-associated molecular patterns and initiate immune responses^[66-69]^. So far, 10 human and 13 murine TLRs have been identified[67]. TLRs are present in different types of cells, including innate immune system components such as macrophages, neutrophils, dendritic cells, NK cells, and mast cells[67]. They are also found in adaptive immune system cells like T and B lymphocytes, as well as stromal cells[67].

In addition to immune cells, TLRs are widely expressed in different types of tumor cells. They regulate tumor growth and function, directly or indirectly, as a “double-edged sword”[66]. They play complex roles in tumor immunity, with either protumor, antitumor, or dual effects[68]. When TLRs interact with a specific molecule, they greatly increase the presence of various signaling molecules on the cell surface, triggering the release of cytokines and the activation of T-cells[67].

Specific TLRs have been implicated in the progression of GI cancers. For example, in esophageal cancer, increased expression of TLR3, TLR4, TLR7, and TLR9 has been linked to esophageal squamous cell carcinomas, while TLR1, TLR2, TLR4, and TLR6 are often overexpressed in esophageal adenocarcinoma[68]. Similarly, in GC, TLR2, TLR3, TLR4, TLR5, TLR7, and TLR9 are all implicated in influencing the advancement of tumors[68]. Notably, increasing expression levels of TLR2, 4, 5, and 9 have been associated with cancer progression from normal gastric mucosa to pre-cancerous lesions, gastric dysplasia, and ultimately to gastric adenocarcinoma[68]. TLRs may have a particular function in the development of GC, as indicated by their increased expression[68]. Another study also found that TLR9 is significantly involved in the progression of GC[69].

TLRs’ capability to propagate immunity makes them attractive targets for the expansion of numerous immunotherapeutic approaches that target cancer[67]. One of the methods involves utilizing TLR ligands/agonists either on their own or as part of combined treatment approaches[67]. These substances are considered immune-boosting elements that amplify TLR signaling and activate an innate immune response resulting in long-lasting adaptive immunity[70]. Several TLR agonists have shown significant efficacy in advanced clinical trials^[67,70]^. For example, dSLIM, a TLR9 agonist, is used for metastatic CRC[70]. Additionally, combination therapy involving TLR9 agonists and ICIs has demonstrated promising effects in clinical studies[67].

TLR-targeted immunotherapy presents a comprehensive approach to treating GI cancers by utilizing the body’s natural immune responses. The various functions of TLRs in both promoting and inhibiting tumors emphasize the importance of understanding their signaling pathways in different cancer scenarios. Ongoing exploration of TLR-mediated mechanisms is essential for developing precise treatments that effectively mobilize the immune system against GI malignancies. The advancing field of TLR-targeted therapies not only shows potential for improving cancer treatment outcomes but also offers valuable insights into the complex relationship between immune responses and tumor biology.

#### Helminth-based immunotherapy

Helminth-based immunotherapy has emerged as a novel approach in the context of GI cancers. For instance, one research indicates that helminth-derived products, such as those from *Taenia crassicjpg*, can enhance the efficacy of conventional treatments like 5-fluorouracil (5FU) by modulating immune responses and promoting apoptosis in tumor cells, particularly in CRC[71]. To illustrate, *T. crassicjpg* molecules have been shown to downregulate pro-inflammatory cytokines and markers associated with malignancy, while promoting NK cell activity and recruitment[71]. In another research study, it was found that mice infected with *T. crassicjpg* had 60% fewer colon tumors compared to uninfected mice[72]. Additionally, 50% of the infected mice did not develop any tumors at all. The impressive outcome was linked to reduced involvement of inflammatory monocytes and the suppression of intensified inflammatory reactions within the colon[72]. Conversely, certain helminths, like *Heligmosomoides polygyrus*, may exacerbate inflammation and tumor development when introduced during the early phases of colitis-associated colon cancer, highlighting the complexity of helminth interactions with cancer pathology[73]. Therefore, the role of helminths in either promoting or delaying the onset of colon cancer has not been fully understood[71].

Given these findings, helminth-based immunotherapy represents a novel and promising approach for treating GI cancers. However, careful consideration is needed due to the complex and context-dependent effects of different helminth species on tumor progression and inflammation.

## Personalized immunotherapy

Previous literature has shown that patients who have the same phenotype of a tumor do not respond the same to a particular immunotherapeutic treatment[74]. This urges that the molecular basis of the tumor needs to be studied for every patient and then allow targeted immunotherapy to be used in such cases. Personalized immunotherapy is very critical as it considers each patient to be an independent group, and this allows every patient to receive a treatment method tailored toward his case only[74]. One of the methods of providing personalized treatment is through classifying the tumors based on mutations in genes like MMR, DNA Polymerase Epsilon, and Rat Sarcoma Viral Oncogene Homolog[74]. This can be done through next-generation sequencing or other methods (bioinformatic technologies[75]) that identify those mutations, especially those that create neoantigens. Neoantigens are novel antigens that are generated by cancer cells due to various causes including genomic mutations[75]. These antigens are classified as non-self and trigger an immune response[75]. Thus, early identification and prediction of these neoantigens[75] can allow for the designing of personalized immunotherapy^[74,75]^, including cancer vaccines[74]. However, this neoantigen-based personalized immunotherapy is currently costly and consumes time to identify the specific neoantigen and assemble the vaccine[74].

For instance, a phase II clinical trial investigated a personalized neoantigen-based peptide vaccine in combination with chemotherapy for the treatment of CRC[76]. The vaccine induced a cellular immune response in 63% of patients and a humoral response in 49%[76]. Moreover, the median OS was 498 days and the 1-year and 2-year survival rates were 53% and 22%, respectively[76]. These results suggest that personalized neoantigen-based peptide vaccines have the potential to improve clinical outcomes for CRC patients.

Personalized immunotherapy is not limited to neoantigen cancer vaccines. Indeed, many other approaches have shown great results such as ACT ones. However, the cost of such treatment remains a barrier. Future research and clinical trials should focus on personalized methods that are cost-effective and that can be performed in a faster time.

## Conclusion

One important treatment for many malignancies, including GI tumors, is immunotherapy. While radiation and chemotherapy remain the mainstays of cancer treatment, immunotherapy is gaining ground as a valuable adjunct therapy due to its potential to target tumors more precisely and with fewer side effects. However, its position as a primary treatment is not yet established because there are still many challenges to overcome. For instance, the tumor’s immunosuppressive microenvironment often limits the effectiveness of immune-based therapies. Future research should aim to address these challenges by investigating how to modulate the tumor microenvironment to allow better immune cell infiltration and activity.

Several key areas of research could significantly advance the field. First, ICIs like anti-PD-1/PD-L1 and anti-CTLA-4 have shown promise but are not universally effective. Future studies should explore combination therapies that can enhance the efficacy of ICIs, such as pairing them with chemotherapy, radiation, or other immunotherapies. Additionally, understanding biomarkers that predict patient responses to ICIs is critical to tailoring treatments to individuals. TLR agonists are another promising area. These molecules can activate innate immune responses and have the potential to be used in combination with ICIs or other immunotherapies to improve outcomes. More research is needed to assess the safety and efficacy of TLR agonists in GI cancers specifically. Helminth-based immunotherapies, while still in the early stages, offer a novel approach by modulating the immune system to suppress inflammation or enhance tumor-fighting responses. Investigating helminth-derived molecules and their ability to work alongside conventional treatments like 5FU should be a focus of future research. Moreover, identifying and validating reliable biomarkers to predict patient responses to various forms of immunotherapy remains a crucial area of study. This will enable more personalized treatments and reduce unnecessary side effects for patients unlikely to respond to certain therapies.

In conclusion, while immunotherapy holds great potential, its future success will depend on further research into combination therapies, overcoming immune resistance, and making the treatments more accessible and affordable. With more focused studies on ICIs, TLR agonists, helminth-based approaches, CAR-T cell therapy, cancer vaccines, oncolytic viruses, and biomarkers, immunotherapy could move from an adjunct therapy to a cornerstone in the fight against GI cancers.

## Data Availability

Not applicable.
